# Pattern and repeatability of ascarid-specific antigen excretion through chicken faeces, and the diagnostic accuracy of coproantigen measurements as compared with McMaster egg counts and plasma and egg yolk antibody measurements in laying hens

**DOI:** 10.1186/s13071-023-05782-5

**Published:** 2023-06-01

**Authors:** Oyekunle John Oladosu, Mark Hennies, Manuel Stehr, Cornelia C. Metges, Matthias Gauly, Gürbüz Daş

**Affiliations:** 1 Research Institute for Farm Animal Biology (FBN), Institute of Nutritional Physiology ‘Oskar Kellner’, Wilhelm-Stahl-Allee 2, 18196 Dummerstorf, Germany; 2 TECOmedical Group, Marie-Curie-Str. 1, 53359 Rheinbach, Germany; 3 Faculty of Science and Technology, Free University of Bozen-Bolzano, Universitätsplatz 5, 39100 Bolzano, Italy

**Keywords:** Diagnosis, ELISA, Helminths, IgY, Nematodes, Repeatability

## Abstract

**Background:**

A coproantigen enzyme-linked immunosorbent assay (ELISA) has recently been proposed for detecting ascarid infections in chickens. The excretion pattern of ascarid antigens through chicken faeces and the consistency of measurements over the course of infections are currently unknown. This study evaluates the pattern and repeatability of worm antigen per gram of faeces (APG) and compares the diagnostic performance of the coproantigen ELISA with a plasma and egg yolk antibody ELISA and McMaster faecal egg counts (M-FEC) at different weeks post-infection (wpi).

**Methods:**

Faecal, blood and egg yolk samples were collected from laying hens that were orally infected with a mix of *Ascaridia galli* and *Heterakis gallinarum* eggs (*N* = 108) or kept as uninfected controls (*N* = 71). Measurements including (a) APG using a coproantigen ELISA, (b) eggs per gram of faeces (EPG) using the McMaster technique and (c) ascarid-specific IgY in plasma and in egg yolks using an ascarid-specific antibody ELISA) were performed between wpi 2 and 18.

**Results:**

Time-dependent significant differences in APG between infected and non-infected laying hens were quantified. At wpi 2 (*t*_(164)_ = 0.66, *P* = 1.00) and 4 (*t*_(164)_ = −3.09, *P* = 0.094) no significant differences were observed between the groups, whereas infected hens had significantly higher levels of APG than controls by wpi 6 (*t*_(164)_ =  −6.74, *P* < 0.001). As indicated by a high overall repeatability estimate of 0.91 (CI = 0.89–0.93), APG could be measured consistently from the same individual. Compared to McMaster and antibody ELISA, coproantigen ELISA showed the highest overall diagnostic performance (area under curve, AUC = 0.93), although the differences were time-dependent. From wpi 6 to 18 coproantigen ELISA had an AUC > 0.95, while plasma IgY ELISA showed the highest diagnostic performance in wpi 2 (AUC = 0.95). M-FEC had the highest correlation with total worm burden, while APG had highest correlations with weights and lengths of *A. galli.*

**Conclusion:**

Ascarid antigen excretion through chicken faeces can be measured with high accuracy and repeatability using a coproantigen ELISA. The antigen excretion increases over time, and is associated with worm maturation, particularly with the size of *A. galli*. Our results suggest the necessity of complementary use of different diagnostic tools for a more accurate diagnosis of infections.

**Graphical Abstract:**

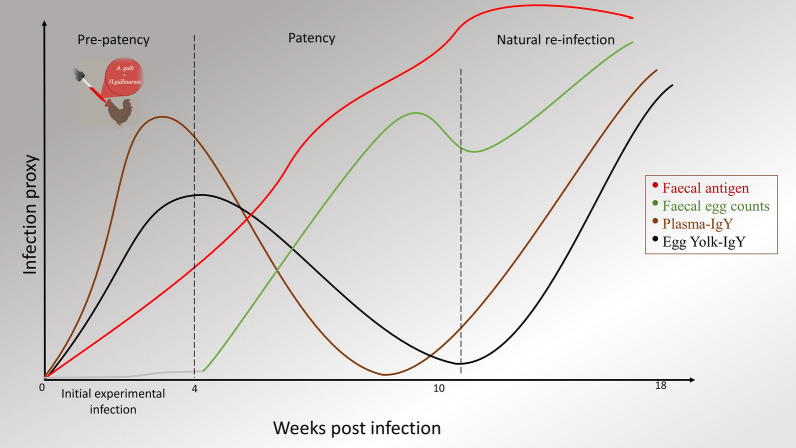

**Supplementary Information:**

The online version contains supplementary material available at 10.1186/s13071-023-05782-5.

## Background

The promotion of practices that improve the welfare of laying hens is increasing the use of non-cage housing systems. When outdoor access is provided, laying hens can better express their natural behaviour and have less fear and stress in a free-range system [1]. As a consequence of keeping hens in non-cage housing systems, gastrointestinal nematodes—in particular *Ascaridia galli* and *Heterakis gallinarum* with oral–faecal transmission routes—have become widespread and are associated with production losses even with minimal or absence of clinical signs [2–7]. In such systems, laying hens are in closer contact with excreta, allowing the oral–faecal transmission of helminth infection [3]. Curbing the spread of the infections and reducing their impact on hen productivity is largely dependent on early and accurate diagnosis.

There are several important criteria to consider when choosing a method of diagnosing helminth infection in livestock. The first is to correctly identify and differentiate all infected and non-infected animals (i.e., qualitative diagnosis). Early detection of helminth infection can prevent the spread of infection within and between flocks. It could also be crucial for employing a targeted flock treatment, which might be cost-effective and could mitigate the development of drug resistance among parasites [8]. The second criterion is for the diagnostic tool to be capable of assessing infection intensity (i.e., quantitative diagnosis) by establishing a significant correlation between its measurement outcome and the actual worm burden of the host animal. Estimating infection intensity is important to poultry and other livestock because the effects of helminth infection on the productivity, health and welfare of animals are likely greater with higher worm burden (e.g., [9]). A quantitative diagnosis is also essential when testing for the efficacy of anthelminthics, which currently relies on the reduction of faecal egg counts (FEC) or worm counts through necropsy [10].

Another criterion for diagnosing helminth infection is to correctly identify specific parasite species causing the infection. However, species-specific identification may not always be a priority in the context of livestock farming, especially because multiple species simultaneously co-infect the host [2, 4, 11], and broad-spectrum anthelminthics are often used in practice to control different intestinal helminths [12]. Under natural conditions, *A. galli* and *H. gallinarum* are known to co-infect the chicken host [11], and a single diagnostic assay is considered sufficient to detect the co-infections by both species [13]. Hence, priority should be accorded to diagnostic methods which have both high qualitative and high quantitative value for assessing nematode infection in chickens.

The gold standard for assessing the intensity of nematode infection is to count the number of worms in different developmental stages in the host intestine, but this requires post-mortem examination [14]. As an indirect method, the presence and intensity of ascarid infection in chickens is generally accomplished by microscopic counting of parasite eggs in faeces, i.e., faecal egg counts (FEC) [15]. McMaster and MiniFLOTAC egg counting techniques are commonly used for quantification of nematode eggs in the faeces of different host species. As shown in two independent studies on chicken ascarids, McMaster is more accurate and faster than MiniFLOTAC, even if the latter has higher precision and sensitivity at low faecal egg counts [16, 17]. Nevertheless, dependence on worm fecundity and infection intensity amongst other challenges can impair the reliability of FECs to assess nematode infections [18, 19]. Therefore, the measurement of ascarid-specific immunoglobulin Y (IgY) in host plasma and egg yolks has been suggested as an alternative diagnostic method to FECs [13, 20]. Recently, we introduced a coproantigen enzyme-linked immunosorbent assay (ELISA) that can quantify soluble ascarid antigens in the faeces of the chicken host with high qualitative diagnostic accuracy [21]. Daş et al. [22] showed that the development of humoral response against *A. galli* in chickens is time-dependent, and larval stages are more strongly associated with antibody stimulation than the adult stages. Such time- and developmental stage-associated changes may also be expected for worm antigen excretion. The test performance of both antibody- and antigen-measuring ELISAs has not yet been compared. Moreover, both ELISAs have so far been separately evaluated only at a single necropsy time point in patent infections [13, 20, 21]. Thus, the evaluations could not account for time-dependent variation in the production of antibodies in plasma or in egg yolks as well as in the excretion of antigens through hen faeces. In addition, there is at present no report on the pattern of worm antigen excretion throughout different phases of infections, i.e., whether worm antigens in host faeces can be consistently measured on the same individual over time.

Therefore, the first objective of this study was to assess the faecal worm antigen excretion pattern over an 18-week period to address time-dependent changes in antigen excretion due to progressing patency and re-infections. This also included estimating the repeatability of faecal antigen concentration measurements in host faeces within and between different weeks post-infection (wpi). The second objective was then to compare the diagnostic performance of the coproantigen ELISA with different diagnostic tools, including faecal egg counts and the measurement of anti-ascaridia antibody in plasma and egg yolk. The ability of the diagnostic methods to estimate the intensity of infection at different wpi was then evaluated.

## Methods

### Ethics statement

The ethics committee for animal experimentation from the Mecklenburg-Western Pomerania State Office for Agriculture, Food Safety and Fisheries, Germany, gave approval for the experiment (permission number AZ.: 7221.3-1-080/16). The experimental procedure for infections followed the guidelines listed by the World Association for the Advancement of Veterinary Parasitology for poultry [23]. Animal handling, care, pen and cage housing, stunning, killing and necropsies were performed by trained and authorized staff members according to the ethical permission and animal welfare rules.

### Experimental design and sample collection

Blood, egg yolk, and faeces samples collected from a total of 179 laying hens of the Lohmann Brown Plus genotype (LB, *N* = 109) and Lohmann Dual genotype (LD, *N* = 70) were used for this study. The laying hens used in this work originated from a previous study [24], where we evaluated the tolerance and resistance of laying hens of different genotypes to nematode infections. The hens were obtained from a research farm (Farm for Education and Research in Ruthe, University of Veterinary Medicine Hannover) as 17-week-old pullets and were randomly allocated into two adjacent rooms each containing six pens. In each room, the hens were kept in three pens per genotype (i.e., three pens for each of the LB and LD genotypes). At the beginning of the experiment, the number of hens kept in the same pen ranged from 8 to 25, with an adjustment for stocking density of a maximum six hens/m^2^. Each hen was given a wing tag to enable repeated measurements on the same individuals over time.

**Fig. 1:**
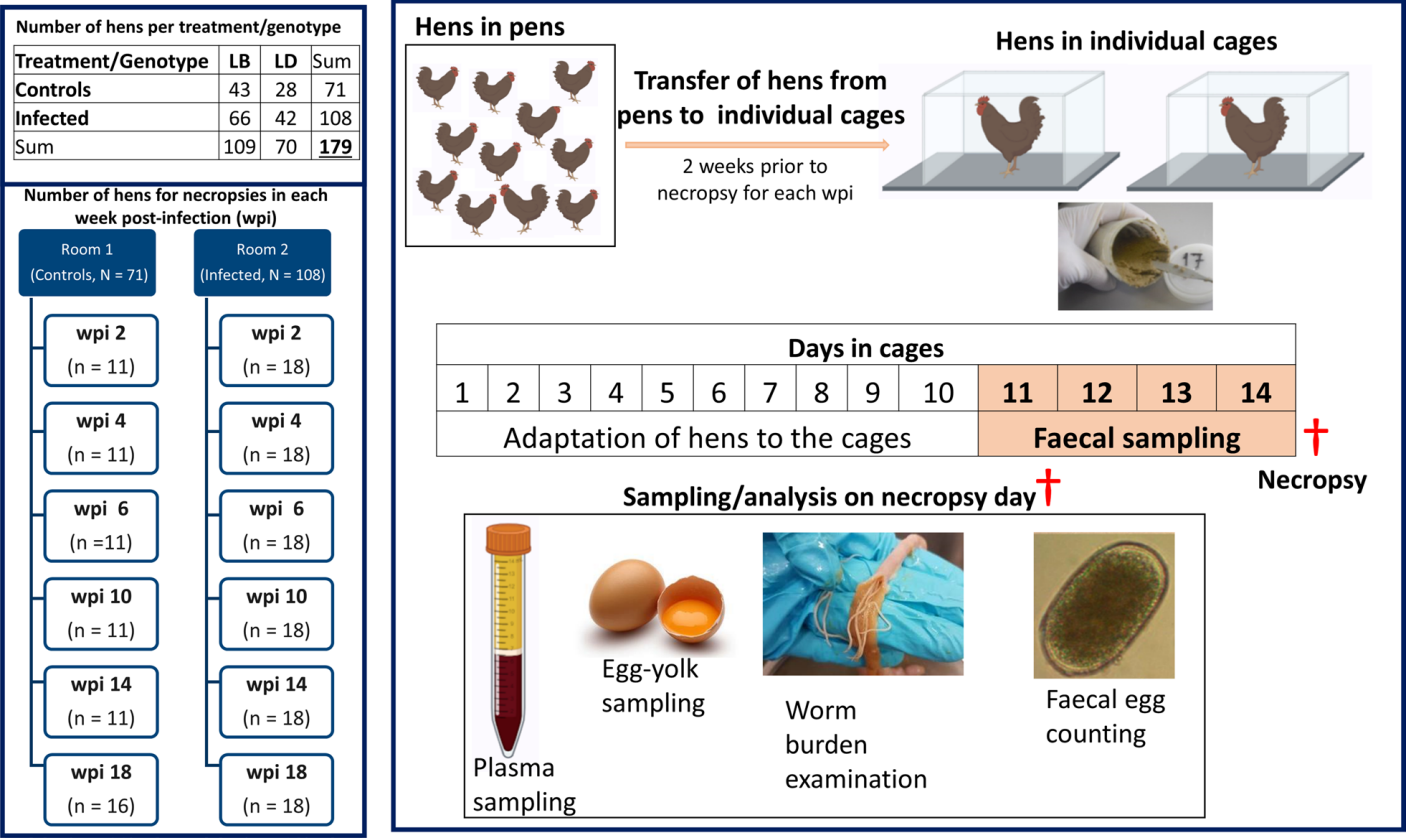
A diagram presenting experimental flow with time-specific necropsies, pen and cage housing schemes, and the number of hens sampled for faeces, blood and egg yolk

At 24 weeks of age, the hens in six pens of the first room (*N* = 108) were experimentally infected with *A. galli* and *H. gallinarum*, while the hens in the six pens of the next room (*N* = 71) were kept as uninfected controls. A consort diagram presenting the number of hens per genotype and infection status, necropsy time points and sampling schemes is given in Fig. 1. At wpi 0, 2, 4, 8, 12 and 16, a total of 29–34 hens were randomly selected from their pens (i.e., all pens were sampled with at least one hen), and transferred to individual cages where they remained for 2 weeks prior to individual egg collection and quantitative faeces sampling (i.e., 24-h sampling). The cages (W 40 × L45 × H 50 cm) with a wire mesh bottom were placed on a faeces collection plate that enabled quantitative daily faeces collection from individual hens. The cages provided equipment for ad libitum water and feed intake of the hens. After an adaptation period of 10 days in the cages, daily individual faeces (g/24 h) were quantified from each hen repeatedly for four consecutive days prior to slaughter. The daily faeces were homogenized thoroughly and subsamples for antigen measurements were stored at −20 °C. After 2 weeks of captivity in the cages, i.e., at wpi 2, 4, 6, 10, 14 and 18, all the caged hens were killed by stunning using a bolt shoot followed by bleeding to death. At each time point, starting from 2 to 14 wpi, 18 infected and 11 control hens were killed for necropsy, while the remaining 18 infected and 16 control hens were killed at wpi 18 (Fig. 1). Immediately after killing, slaughter blood was collected in potassium-ethylenediaminetetraacetic acid (EDTA)-treated tubes (Kabe Labortechnik GmbH, Nümbrecht-Elsenroth, Germany), and the hens were necropsied to assess worm burden as a direct measure of infection intensity. The blood samples were centrifuged for 20 min at 2500×*g*, and the resulting supernatant was stored at −20 °C for later analysis. Individual eggs were collected from each hen during the last day of captivity or at slaughter. Sampled eggs were opened to collect the egg yolks. A subsample of egg yolk (250 µL) was collected and diluted with 1.5 ml of purified water (pH = 2.5) and homogenized using a vortex mixer. The egg yolks were stored at −20 °C until analysis. A total of 179 blood and egg yolk samples and 716 (i.e., 179 hens × 4 days) faecal samples were therefore recorded from all laying hens throughout the experimental period.

Wood shavings were used as the litter materials in the pens. On the day of the infection, the litter was renewed and thereafter left in the pen for 18 weeks to allow subsequent natural infection to occur. All hens were fed a commercial diet (ad libitum), containing 11.2 MJ metabolizable energy, 170 g crude protein and 3.6 g calcium/kg feed for laying hens [24]. The climatic conditions in the rooms were controlled using an automatic system to ensure constant temperature, light and aeration across all the pens.

### Experimental infection procedures and measurements of infection intensity

Infection material for the experiment was collected from female worms residing in the intestines of free-range hens that were naturally infected with ascarids. The procedure for worm recovery and isolation from chicken intestines and the embryonation of eggs of *A. galli* and *H. gallinarum* have been described previously [25]. For the embryonation of *A. galli* eggs, 0.1% potassium dichromate (K_2_Cr_2_O_7_) was used as the incubation medium, whereas intact *H. gallinarum* females were kept in formalin (0.5%) at room temperature for approximately 4 weeks. Eggs of *H. gallinarum* were isolated from the worms 1 day prior to infection, as described in Stehr et al. [25]. For the preparation of infection material to be given to the hens, the embryonated eggs of both species were rinsed in a 36-µm sieve and collected in 0.9% NaCl in two separate egg pools (i.e., for *A. galli* and *H. gallinarum* separately) at room temperature. The egg pools were assessed to determine the percentage of eggs that were fully embryonated [26]. After adjustment of the concentrations of the embryonated eggs in the 0.9% NaCl solution (i.e., 500 eggs of *A. galli* or *H. gallinarum* in 0.2 ml NaCl), a total of 1000 embryonated eggs of both species in 0.4 ml of NaCl was given to each hen. The hens were inoculated with the infective eggs of the two parasites using a 5-cm oesophageal cannula in a single dose (i.e., 500 *A. galli* + 500 *H. gallinarum* eggs). The uninfected control hens were given an oral placebo containing 0.4 ml of 0.9% NaCl.

#### Worm burden

Worm burden was quantified from laying hens that were necropsied at wpi 2, 4, 6, 10, 14 and 18. The hens were subjected to fasting for 3 h prior to necropsy to partly empty the gastrointestinal tract (GIT). The GIT was removed immediately after the necropsy, and the small intestine and caecum, the predilection sites of *A. galli* and *H. gallinarum*, respectively, were separated [24]. The jejunum and ileum were then opened longitudinally to wash the intestinal contents through a sieve (mesh size: 36 μm and 100 μm at 2–6 wpi and 10–18 wpi, respectively). After removal of intestinal content, the jejunum was rinsed under running lukewarm tap water while simultaneously squeezing the tissue through a pair of fine pencil-pincers to remove the lumen worms attached to the tissue walls. Tissue larva recovery was done only in the jejunum using an EDTA incubation [27]. Briefly, the jejunum was incubated in 400 ml of preheated EDTA solution (10 mM EDTA, 0.9% NaCl) for 22 h at 40 °C. After incubation, the tissue was dipped in EDTA solution to remove the larvae. The solution was sieved through a sieve (mesh size: 20 μm) to collect the tissue larvae.

All recovered worms of both species from each hen were placed in Petri dishes for counting, sex differentiation and length measurements using a stereomicroscope. The total number of each of the *A. galli* and *H. gallinarum* worms present in the small intestine and caeca were recorded separately. Worm burden was recorded based on the identified morphological characteristics of worms such as sex (male or female) and by developmental stage (i.e., larvae, mature, immature) [25]. Briefly, identification of male worms was based on the presence of spicules. Worms of *H. gallinarum* were classified as adult females if eggs were present in the uterus. *Ascaridia galli* worms were classified using a predetermined cut-off (43.5 mm) to precisely separate ovigerous females (> 43.5 mm) from immature females (< 43.5 mm) [25]. Worm length was measured for both *A. galli* and *H. gallinarum* using a ruler with measurement precision of 1 mm. Length measurement was based on the worm classification (i.e., larvae, mature immature, males). Only intact worms for each classification (maximum of 10 per bird) were randomly selected and measured. The average of the selected worms was multiplied by the total number of worms in each classification [25]. The weight (mg) of *A. galli* was estimated from the length (mm) of female and male *A. galli* using the Le cren weight–length relationship model [28]. The average weight of *A. galli* was also calculated with respect to the total *A. galli* burden in each hen. To establish the Le cren weight–length relationship, we used a data set from a previous experiment (Additional file 1: Fig. S1), where both the weight and length of *A. galli* were precisely measured. For the measurement of *A. galli* weight, a precision (0.1 mg readability) analytical balance (Mettler Toledo GmbH, Gießen, Germany) was used. The precision of length measurements was the same as in the present study (i.e., 1 mm).

#### Faecal egg counts (FEC)

At each time point of necropsies (i.e., wpi 2–18), a random subsample (4 g) was obtained from thoroughly mixed daily faeces collected 1 day prior to hen necropsy and analysed with the McMaster egg counting technique [15]. A saturated NaCl solution (density = 1.2 g/ml) was used as the flotation fluid for the 4 g of faeces, which was then made up to 60 ml of the final suspension. The minimum detection level of the egg counting technique was set to 50 eggs per gram of faeces (EPG). Since eggs of *A. galli* and *H. gallinarum* cannot reliably be differentiated from each other [29], and regular faecal droppings cannot be precisely separated from the caecal droppings following a 24-h collection period, both regular and caecal droppings were mixed, and ascarid eggs were counted together.

#### Quantification of ascarid-specific antigens in faeces and ascarid-specific IgY in plasma and egg yolk

Homogenized faecal subsamples taken from the daily faeces of hens during the last 4 days of captivity (i.e., *n* = 4 samples/hen) were measured for worm antigen concentration according to the ELISA procedure described by Oladosu et al. [21]. Briefly, soluble antigens from *A. galli* were isolated from thawed intact worms by washing with phosphate-buffered saline (PBS) and 70% ethanol. Afterwards, the worms were homogenized in a mortar and extracted using basic buffer (35 mM BisTris, 25 mM Tris). Extracted soluble antigen was used to immunize rabbits for the production of antibody. ELISA plates were coated overnight at 4 °C with 100 µl of the anti-ascarid polyclonal rabbit antibody to allow binding with soluble antigen in faecal samples. A total of 50 mg of daily faecal samples was weighed in sample buffer and mixed thoroughly with a vortex mixer. The supernatant was collected and pipetted into assay wells. An aliquot of 100 µl of soluble antigen with concentrations of 400, 200, 100, 50, 25 and 0 ng/ml were also added to the assay wells for standardization. The plates were then incubated and washed repeatedly before measurements. The antigen concentration in faeces was then measured as the amount of ascarid antigen per gram of faeces (APG, µg/g faeces) based on the standard curve for 400, 200, 100, 50, 25 and 0 ng soluble antigen/ml [21]. Anti-ascarid-specific IgY in plasma and egg yolk was quantified using another ELISA [13]. The microtiter plates were coated overnight at 4 °C with 100 µl of the isolated soluble *A. galli* antigen. Standard chicken serum was serially diluted and used as the standard curve in the assay. Samples were added to the coated plates and incubated for 2 h. After incubation, the plates were washed repeatedly followed by another 30 min incubation with enzyme conjugate, washing step and termination with hydrochloric acid. Antibody binding was expressed relative to the standard chicken serum with high antibody activity (1000 mU/ml per definition) using a four-parameter logistic (4-PL) [13].

### Statistical analyses

Data were modelled based on the measurement of each variable, i.e., either single measurements or repeated measurements from a host over time. The relevant worm burden parameters (worm counts, worm length, FEC, etc.) were measured at a single time point during necropsy, while antigen concentration was measured in each of the faecal samples collected on four consecutive days prior to necropsy (Fig. 1). APG, egg yolk IgY and plasma IgY data were analysed after log transformation [log(*y*+1)] to correct for the heterogeneity of variance and produce approximately normally distributed data. A description of all the variables measured is presented in Table 1.

**Table 1:** Abbreviation, measurement unit, and short description of relevant variables

Abbreviation	Unit	Description
APG	µg/g faeces	Antigen per gram of faeces
Ag_Length	cm	*A. galli* length
Hg_Length	mm	*H. gallinarum* length
EPG	*n*/g faeces	Number of eggs per gram of faeces
Ag_Total	*n*/hen	Total number of *A. galli* in a hen
Hg_Total	*n*/hen	Total number of *H. gallinarum* in a hen
Ag_Weight	mg/hen	Average weight of all *A. galli* in a hen
Egg yolk_IgY	mU/mL	Ascarid-specific IgY in egg yolk
Plasma_IgY	mU/mL	Ascarid-specific IgY in plasma

Significant differences in the antigen and antibody concentrations between infected hens and non-infected control within wpi and their interactions were analysed with repeated-measures analysis of variance (ANOVA) using the PROC MIXED function of the SAS OnDemand for Academics cloud-based software (2021 SAS Institute Inc., Cary, NC, USA). The repeated statement was excluded for antibody concentration in plasma and egg yolk since only a single measurement at necropsy was done. The model for antigen and antibody concentrations included the fixed effects of infection, wpi and their interactions, while pen, host genotype and sampling day were considered as blocking effects in the analysis. The covariance structure was set to AR (autoregressive) (1) for the fitted model. Least-square means were computed for each fixed effect, and pairwise comparison was tested with the Tukey–Kramer corrections for multiple comparisons. Effects and differences were considered significant at *P* < 0.05.

The intra-class correlation coefficient (ICC) between the repeatedly measured samples within each wpi was estimated to determine the repeatability of faecal antigen excretion. Faecal samples from both infection groups for quantifying antigen concentration were repeatedly collected from the same laying hens for each of four consecutive days (*n* = 4 samples per hen) in each wpi (*n* = 4 × 29–34 samples per wpi). ICC estimates of these four repeated measurements and their 95% confidence intervals (CI) were calculated using the ICC function in the R psych package version 2.1.9 [30]. Estimates were based on the absolute agreement of measurements (*k* = *4*), two-way mixed-effects model [31]. Measurements with ICC values of less than 0.5 were defined as having poor reliability, values between 0.5 and 0.7 as moderate reliability, values between 0.75 and 0.9 as good reliability, and values greater than 0.9 as excellent reliability [31].

A receiver operating characteristic (ROC) analysis was performed to assess and compare the diagnostic accuracy of coproantigen ELISA with that of FEC, plasma, and egg yolk IgY ELISA using samples collected 1 day prior to slaughter. The pairwise comparison of the area under the curve (AUC) from all diagnostic tests was carried out using the DeLong post hoc test [32] because the faecal, egg yolk and blood samples used for the FEC, coproantigen and the IgY measurements were made on the same host. Test accuracy of the assays was interpreted based on the range of the AUC value and is classified as follows: low accuracy (0.5 < AUC ≤ 0.7), moderate accuracy (0.7 < AUC ≤ 0.9) or high accuracy (AUC > 0.90) [33].

The data sets used for ROC comparison included APG, EPG, plasma, and egg yolk IgY values of experimentally infected laying hens with their corresponding controls obtained during necropsy. The analysis was performed for both data pooled across wpi and within wpi to determine differences between different time points, i.e., potential time-dependent differences in overall performance of different tests. In any case, the total number of observations (*n* ≥ 28) used for the analysis exceeded the minimum sample size required for an ROC analysis. The minimum sample size was calculated using MedCalc statistical software version 20.023 [34] with a preset significance level of 0.05, maximum AUC of 0.99 and group ratio of 1 [33]. All parameters of the ROC and DeLong comparison test were calculated using the pROC open-source package for R [35].

Pearson correlation coefficients were calculated to determine the interdependence among infection-related parameters including worm burden, worm length, worm weight, FECs and antigen concentration in faeces. The analysis was based on log-transformed data [log(*y*+1)]. To further assess the quality of each diagnostic method, Pearson correlations among all variables (i.e., ascarid-specific IgY in plasma and egg yolk, APG and EPG with worm burden parameters) were examined within each wpi. Pearson correlation analysis, descriptive statistics and visualization of data were performed in the R environment for statistical computing [36].

## Results

### Ascarid antigen concentration in host faeces

**Fig. 2:**
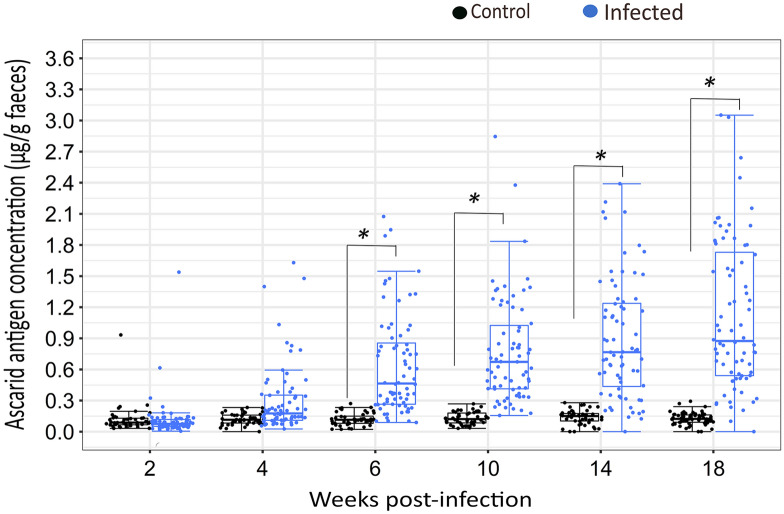
Concentration of ascarid antigens in faeces (APG) of control (black circle) and infected (blue circle) laying hens. The effect of infection, wpi and their interaction were significant (*P* < 0.001). Statistical analyses are based on log-transformed data [log(y+1)] while visualization is based on untransformed data. * Indicates a significant difference between control and infected laying hens at a given time point (Tukey–Kramer, *P* < 0.05). Each dot on the plot represents an individual observation. Number of observations (*N* = 716) refers to 179 hens sampled for four consecutive days prior to slaughter. The vertical line inside the box plots shows the sample median, while the lower and upper ends of the box represent the 25th and 75th quantiles, respectively

The fixed effects of infection (*F*_(1,164)_ = 211.05, *P* < 0.001), wpi (*F*_(5,164)_ = 18.60, *P* < 0.001) and their interactions (*F*_(5,164)_ = 15.85, *P* < 0.001) on antigen concentration (APG) were significant (Table 2). Tukey–Kramer adjusted pairwise comparison for within-wpi effect revealed that antigen concentration was not significantly different between control and infected animals at wpi 2(*t*_(164)_, = 0.66, *P* = 1.00) and wpi 4 (*t*_(164)_, =  −3.09, *P* = 0.094) (Fig. 2). APG increased in infected laying hens by wpi 6 (*t*_(164)_, =  −6.74, *P* < 0.001), and significant differences (*P* < 0.001) were then observed between infected and uninfected laying hens until the end of the experiment at wpi 18. Control hens were not significantly different (post hoc, Tukey–Kramer adjusted *P* > 0.05) in their antigen concentration across different wpi throughout the experiment (Fig. 2), whereas there was a statistically significant increase (*P* < 0.05) in the APG values of infected laying hens across the wpi. APG in the infected laying hens was significantly different (*t*_(164)_, =  −4.62, *P* < 0.001) in the early stages of infection (between wpi 2 and 4), while at later phases (e.g., between wpi 6–18, [*t*_(164)_, =  −3.09, *P* = 0.094]) there was no significant difference in the APG of infected laying hens.

**Table 2:** Effects of mixed-nematode infection on ascarid-specific IgY in plasma and egg yolk and the concentration of ascarid antigens in faeces of uninfected control or infected chickens

Variables	Infection status				Statistics								
	Control		Infected		Infection			WPI			Infection × WPI		
	LSM	SE	LSM	SE	*P*	*df*	*F*	*P*	*df*	*F*	*P*	*df*	*F*
Plasma IgY (mU/mL)	23.5	10.75	86.3	8.91	0.001	164	82.85	0.131	164	1.73	0.001	164	4.56
Egg yolk IgY (mU/mL)	3.77	7.81	41.94	6.53	0.001	151	95.38	0.009	151	3.19	0.008	151	3.26
APG (µg/g faeces)	0.13	0.04	0.65	0.03	0.001	164	211.05	0.001	164	18.6	0.001	164	15.85

Statistical analyses of the data are based on log-transformed data [log(*y*+1)]. whereas least-squares means (LSM) and their standard errors (SE) are based on untransformed data

APG was quantified from birds on four consecutive days prior to necropsy in each week. Thus, the number of observations used for the statistical analysis was *N* = 179 hens × 4 days = 716 APG measurements

The data used for the analysis of plasma and egg yolk IgY are based on single measurements at necropsy in each wpi (*n* = 179)

*APG* ascarid antigen concentration per gram of faeces, *IgY* ascarid-specific IgY in plasma or egg yolk, *P* *P*-value, *df* degrees of freedom, *F* *F*-value

### Repeatability of worm antigen excretion through host faeces

**Fig. 3:**
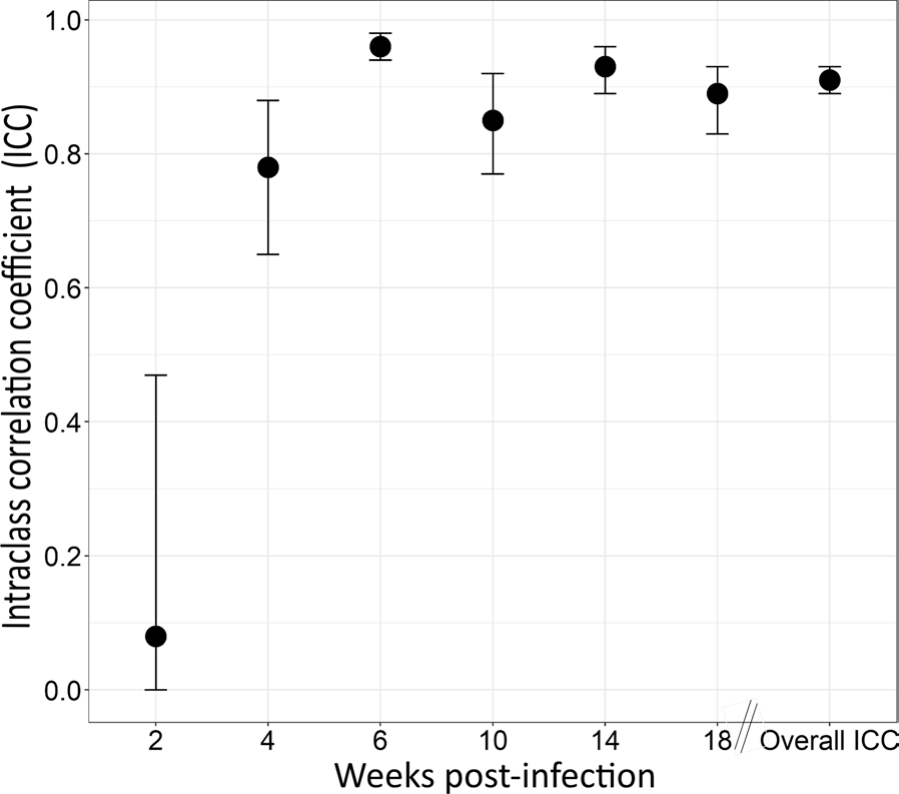
Intra-class correlation coefficient (ICC) of repeated measurements of antigen concentration per gram of faeces within each week post-infection (wpi) and the overall ICC. Number of hens necropsied, *n* = 29 in each wpi, and in wpi 18, *n* = 34 (overall *N* = 179 hens). Error bars represent 95% confidence intervals

The ICC estimates of the repeated measure of APG are given in Fig. 3. Overall, the analysis revealed a high repeatability estimate (ICC = 0.91; 95% CI = 0.89–0.93) for APG measurement from the same animal across four repeated measurements within a wpi. There were fluctuations, however, in the repeatability of APG across different weeks. The ICC estimates for the measurements in wpi 2 were low (ICC = 0.08; 95% CI = 0–0.47), but APG measurements throughout the remaining wpi showed moderate to high repeatability estimates (ICC 0.78–0.96). The highest repeatability estimate (ICC = 0.96; 95%CI = 0.94–0.98) was recorded in measurements obtained in wpi 6.

### Ascarid-specific IgY in host plasma and egg yolks

**Fig. 4:**
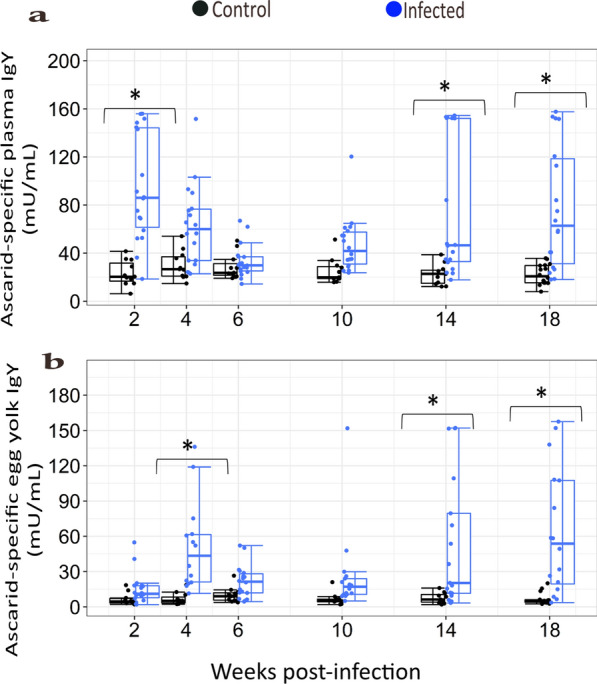
Ascarid-specific IgY concentrations in **a** plasma and **b** egg yolks of control (black circle) and ascarid-infected laying hens (blue circle). The effect of infection and interaction with wpi were significant (*P* < 0.001). Statistical analyses are based on log-transformed data [log(*y*+1)] while visualization is based on untransformed data (*n* = 179 hens). * Indicates a significant difference between control and infected laying hens at a given time point (Tukey–Kramer, *P* < 0.05). Each dot on the box plot represents an individual observation. The vertical line inside the box plots shows the sample median, while the lower and upper ends of the box represent the 25th and 75th quantiles, respectively

A statistically significant difference was found in overall ascarid-specific IgY concentration in both plasma (*F*_(1,164)_ = 82.85, *P* < 0.001) and egg yolk (*F*_(1,151)_ = 95.38, *P* < 0.001) between infected and uninfected controls (Table 2). The overall average IgY concentration across all wpi in infected laying hens was higher than that in uninfected controls. However, the differences were time-dependent. The differences in the antibody response in plasma between the two groups were significant at wpi 2 (*t*_(164)_ = −6.35, *P* < 0.001), wpi 14 (*t*_(164)_ = −5.05, *P* < 0.001) and wpi 18 (*t*_(164)_ = −5.37, *P* < 0.001) (Fig. 4a), while significant differences in egg yolk IgY concentrations were observed at wpi 4 (*t*_(151)_ = −5.78, *P* < 0.001), wpi 14 (*t*_(151)_ = −4.67, *P* = 0.0004) and wpi 18 (*t*_(151)_ =  −6.29, *P* < 0.001) (Fig. 4b).

### Worm burden and FEC

**Fig. 5:**
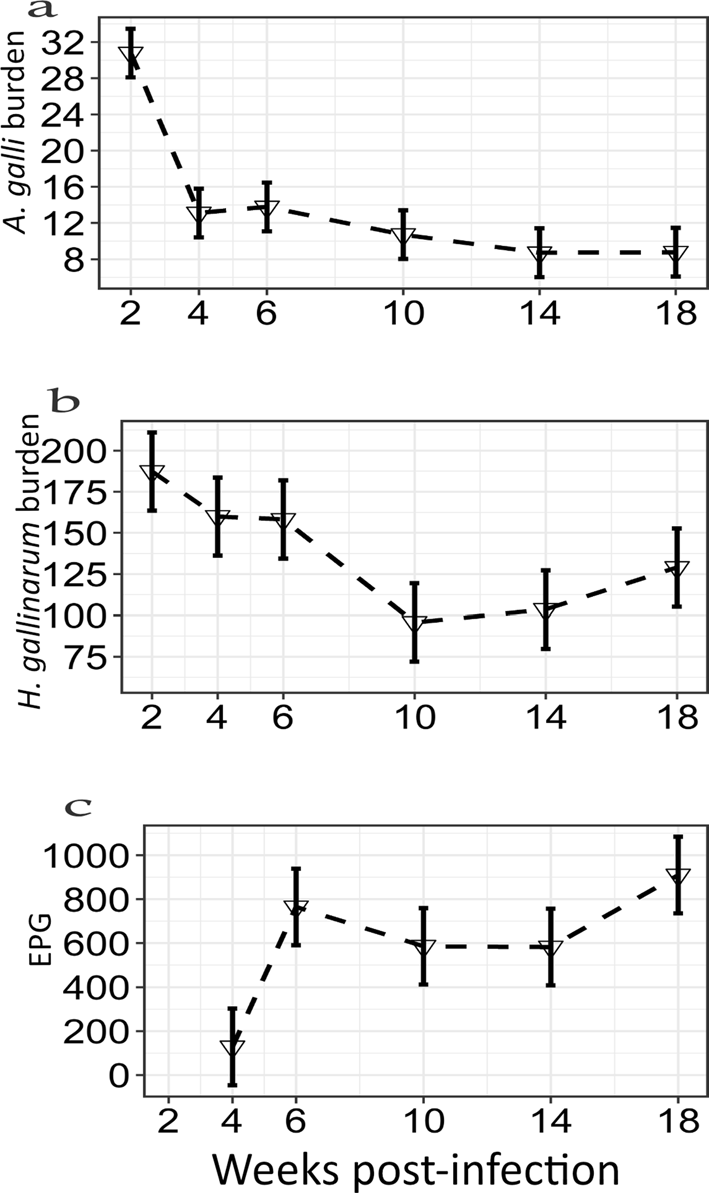
Worm burden of laying hens experimentally infected with both *Ascaridia* *galli* (**a**) and *Heterakis* *gallinarum* (**b**), and the number of eggs per gram of faeces (EPG) (**c**) across different weeks post-infection (*n* = 108). Figures are LS means with their standard errors. The EPG was determined by wpi 4. Thus, the number of faecal samples for EPG was *n* = 90

A detailed presentation of worm burden in both host genotypes has been reported previously [24]. Figure 5 provides visual representations of the worm burden of the hens with *A. galli* and *H. gallinarum* as well as FEC resulting from both nematodes. The number of worms was highest at wpi 2 for both *A. galli* (Fig. 5a) and *H. gallinarum* (Fig. 5b). The number of *A. galli* worms decreased over time throughout the experimental period such that the lowest number of worms was recovered by wpi 18, whereas the lowest count of *H. gallinarum* was recovered in wpi 10. The number of *H. gallinarum* increased from wpi 14 through wpi 18 due to re-infections. EPG was not quantified until wpi 4 (Fig. 5c). The average EPG increased between wpi 4 and wpi 6 and then remained relatively constant until wpi 14, while the highest average EPG was observed at the last wpi. Worm eggs were not present in the faeces of control laying hens throughout the period of the experiment.

### Diagnostic accuracy of coproantigen ELISA, antibody ELISA and FEC

**Fig. 6:**
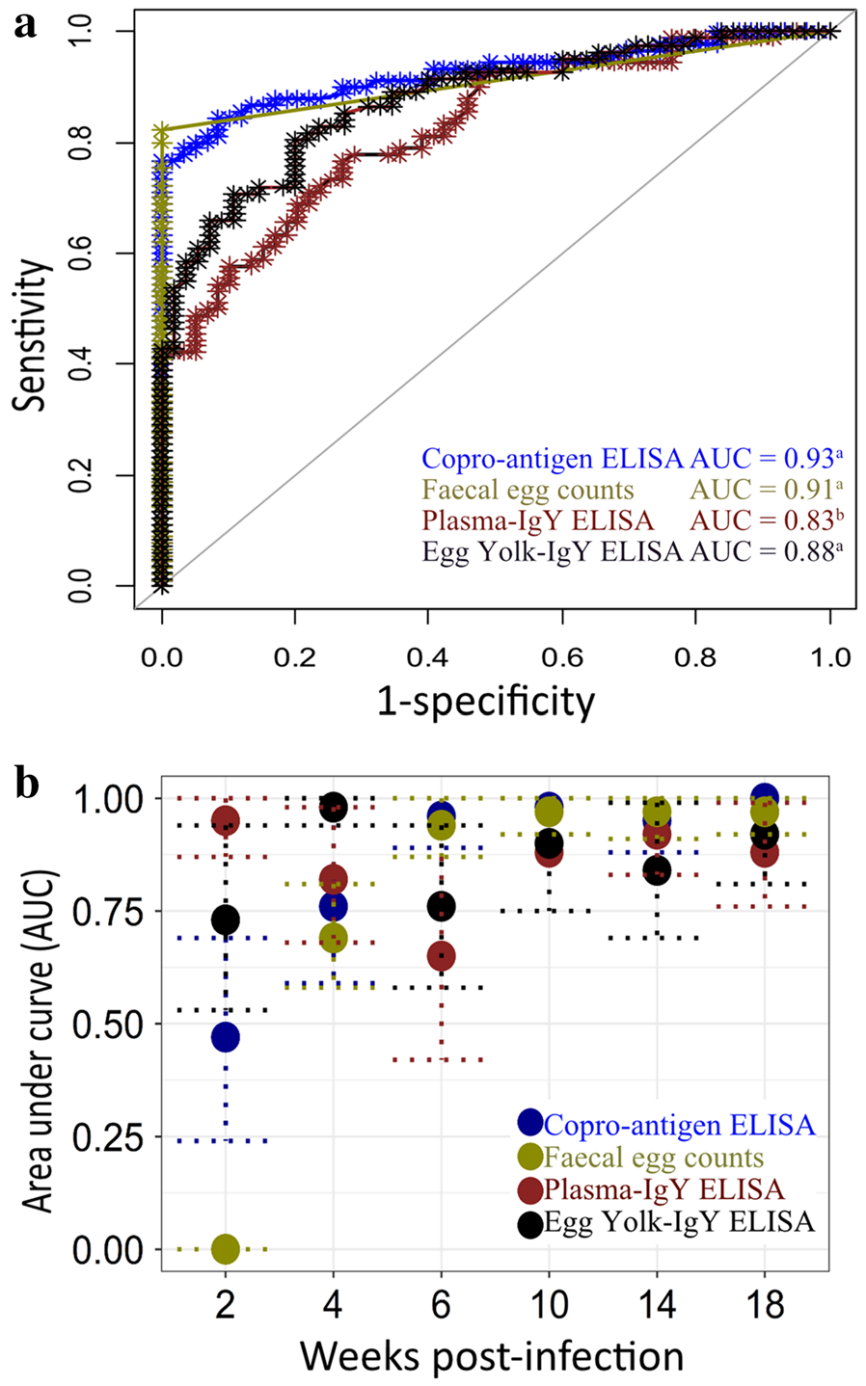
Overall (**a**) and time-point-specific (**b**) diagnostic accuracy of the coproantigen ELISA in comparison with plasma and egg yolk IgY ELISA and faecal egg counts in chickens infected with *Ascaridia* *galli* and *Heterakis* *gallinarum*. The diagonal line corresponds to half of the maximum area under the curve (AUC = 1.0). The DeLong test was used to determine significant differences between the AUC of each diagnostic test. The significance level was preset at *P* < 0.05. AUC values bearing the same superscripts showed no significant difference. The farther the location of the ROC curve from the diagonal line, the higher the total test accuracy analyses. A summary of AUC values with their confidence intervals for all diagnostic methods within each wpi is shown in (**b**)

ROC analysis was carried out to investigate the diagnostic accuracy of the coproantigen ELISA compared with FEC, egg yolk and plasma IgY ELISA using all measurements taken within each wpi during the experiment. The outcomes of the ROC analysis are summarized in Fig. 6, and detailed results with specific test performance parameters (e.g., AUC, sensitivity, specificity) are presented in Additional file 2: Table S1. The overall accuracy for coproantigen ELISA was high, with AUC = 0.93. Except for wpi 2 and 4, high accuracy (AUC > 0.90) was confirmed for this method within all wpi. Its diagnostic test accuracy was highest at wpi 18 (AUC = 1.00). Similarly, the specificity and sensitivity of the assay increased over time; by wpi 4, the assay yielded 100% specificity but with low sensitivity of 39%. To fully compare the accuracy of the coproantigen ELISA method (i.e., APG) with faecal egg counts (i.e., EPG) and plasma and egg yolk IgY ELISA, the available data for EPG and IgY assay were used. The overall AUC for FEC was 0.91, while plasma and egg yolk IgY assay yielded overall accuracy of AUC = 0.83 and 0.88, respectively (Fig. 5a). The DeLong test showed no significant difference between coproantigen ELISA and FEC (*Z* = 0.674, *P* = 0.501) and egg yolk IgY ELISA (*Z* = 1.645, *P* = 0.100), whereas the overall accuracy of plasma IgY assay was significantly (*Z* = 2.336, *P* = 0.019) lower. Both FEC and coproantigen ELISA had 100% specificity, while specificity was lower for the plasma IgY assay (72.9%) and egg yolk IgY assay (80%). FEC demonstrated the highest sensitivity, at 82.2%, followed by egg yolk IgY with sensitivity of 80%, while both coproantigen and plasma IgY ELISA had sensitivity of 76.7%.

### Correlations among infection-related parameters

**Fig. 7:**
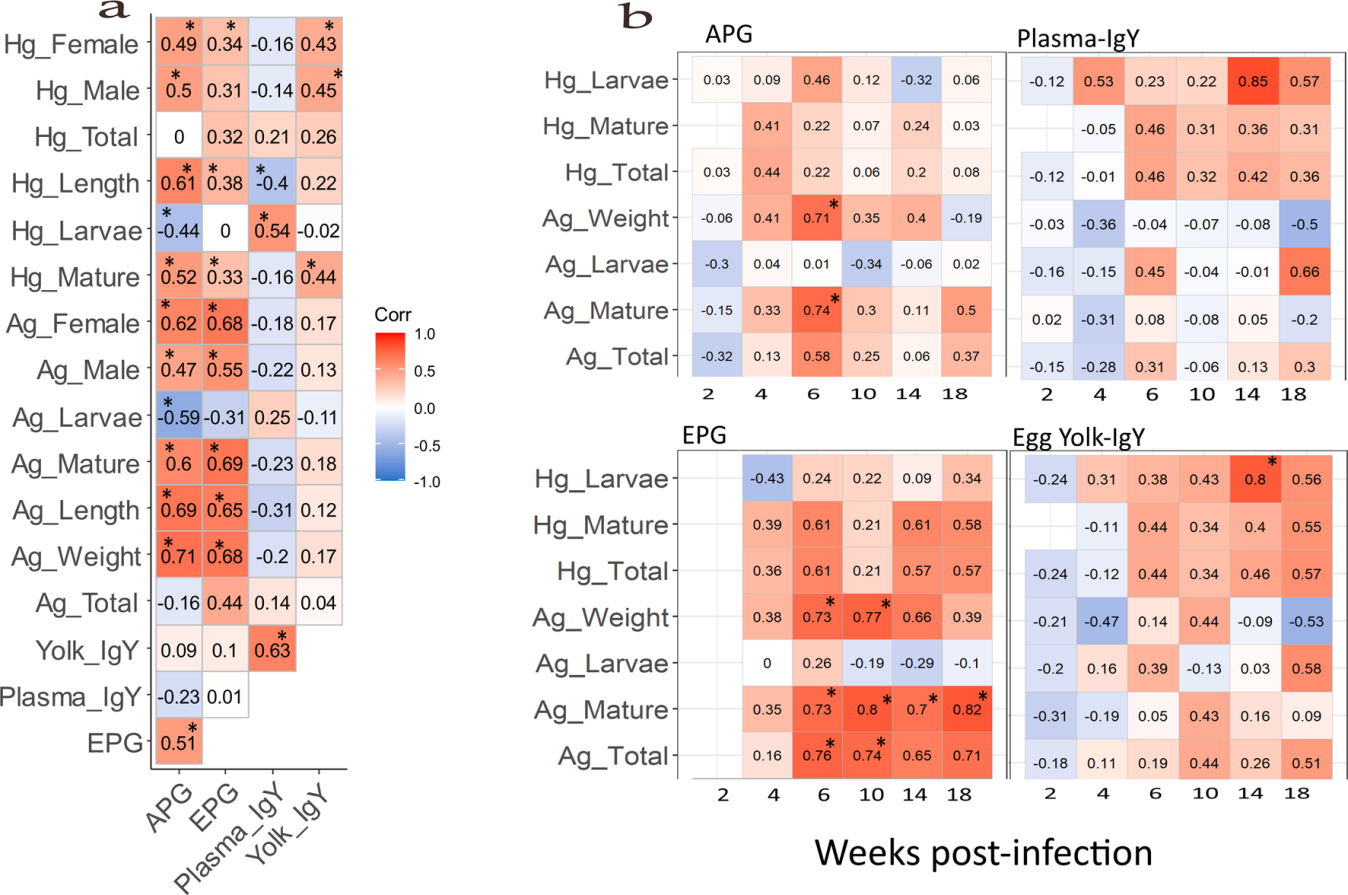
Overall correlations with pooled data across wpi among different variables representing worm burden and diagnostic measurements (**a**), and Pearson correlation coefficients between each worm burden variable and APG, EPG and IgY within each wpi (**b**) (*n* ≥ 29). The Pearson correlation coefficients (*r*) are presented in the squares. Significant (*P* < 0.05) correlations are indicated with an asterisk (*)

We investigated the linear relationships among all infection-related parameters using Pearson correlation statistics with both pooled data across all the weeks (Fig. 7a) and within each wpi (Fig. 7b) by each diagnostic measurement. The parameters associated with the length and weight of the worms demonstrated higher positive correlation coefficients with APG than EPG and both IgY. The total average length of *A. galli* showed a significant positive correlation with APG (*r*_(105)_ = 0.69, *P* < 0.001) and EPG (*r*_(87)_ = 0.65, *P* < 0.001). In the case of *H. gallinarum*, the total worm length demonstrated significant positive correlations with APG (*r*_(102)_ = 0.61, *P* < 0.001) and EPG (*r*_(84)_ = 0.38, *P* = 0.030). However, a negative correlation was found between plasma IgY and the total length of both *A. galli* (*r*_(105)_ =  −0.31, *P* = 0.100) and *H. gallinarum* (*r*_(102)_ =  −0.40, *P* = 0.010). The weight of *A. galli* showed a significant correlation (*r*_(105)_ = 0.71, *P* < 0.001) with APG and EPG (*r*_(87)_ = 0.68, *P* < 0.001).

Only EPG exhibited a high correlation with the total worm counts of both *A. galli* (*r*_(87)_ = 0.44, *P* < 0.001) and *H. gallinarum* (*r*_(87)_ = 0.32, *P* = 0.110). However, when we examined the correlation with the number of worms by maturity, there was a significant positive correlation between APG and the total number of adult worms (*r*_(105)_ = 0.6, *P* < 0.001 for *A. galli* and *r*_(105)_ = 0.52, *P* < 0.001 for *H. gallinarum*), and a negative correlation with the total number of larvae (*r*_(105)_ = −0.59, *P* < 0.001 for *A. galli* and *r*_(105)_ = −0.44, *P* < 0.001 for *H. gallinarum*). EPG correlated negatively with the *A. galli* larvae (*r*_(87)_ = −0.31, *P* = 0.160) and positively with mature worms (*r*_(87)_ = 0.69, *P* < 0.001 with *A. galli* and *r*_(87)_ = 0.33, *P* = 0.070 with *H. gallinarum*). Plasma IgY demonstrated a positive correlation with *H. gallinarum* larvae (*r*_(105)_ = 0.54, *P* < 0.001) and *A. galli* larvae (*r*_(105)_ = 0.25, *P* = 0.370) but egg yolk IgY did not. We further investigated whether the correlations between worm burden and infection proxies are time-dependent. The result in Fig. 7b shows that the highest correlation occurred at different wpi for each of the four infection proxies. Correlations were lowest at wpi 2 for all methods. In general, EPG showed significant positive correlations with worm counts and size measurements in most wpi (Fig. 7b; Panel EPG).

## Discussion

This study assessed the excretion pattern and repeatability of ascarid antigen in the faeces of laying hens over time and evaluated the performance of four different methods of diagnosing nematode infections, with an emphasis on alterations in test performance at different time points of infection. The methods included McMaster faecal egg counts, coproantigen ELISA, and plasma IgY and egg yolk IgY ELISA. It is evident from the results that soluble worm antigens are rather consistently excreted through the faeces of laying hens. Antigen concentrations increased over time in the faeces of infected hens, indicating the possibility that as worms mature, antigen concentration increases. In a time-dependent manner, increased antigen concentration may also be a reflection of higher worm burden and re-infection.

To confirm whether APG can be consistently measured on the same hen over time, we performed repeatability estimates of antigen excretion by measuring the antigen concentration from faecal samples of the same individual hens for four consecutive days within a given wpi. Repeatability is the measure that reflects the extent to which a set of measurements can reliably be replicated by measuring both the correlation and absolute agreement among the obtained values [37]. The overall repeatability (ICC = 0.91) confirms a considerable repetitive pattern in the excretion of antigens in faeces. From wpi 4, when antigen could be reliably quantified, the agreement among measurements across the four sampling days was high, suggesting that antigen excretion in faeces of the infected hens consistently occurred daily and is reliably measurable on the same animal.

Furthermore, the results also showed unique time-dependent differences in the test performance of different diagnostic methods, implying the need for specific selection of one or two tools to capture a more informative indication of nematode infections in chickens. Faecal egg counts are widely used for assessing *A. galli* and *H. gallinarum* infections, but due to challenges such as variability in worm fecundity, diurnal variation in excretion of eggs and uneven distribution of eggs in faeces [19, 38], new methods have been investigated. This includes measuring worm-specific antibody in host plasma and egg yolks [13, 20] and soluble worm antigens in host faeces [21], as well as PCR-based approaches [39]. Ascarid-specific plasma IgY was already significantly different between infected and non-infected hens at wpi 2, which is in agreement with all relevant past studies measuring immune response to nematode infection in chickens [22, 40–42]. We deduce that diagnosis with antibody ELISA can provide earlier detection of infection than faecal egg counting or coproantigen ELISA. *Heterakis gallinarum* larvae are carried to caeca nearly 9 h after the host’s ingestion of the ova, where the larvae embed themselves into the superficial epithelium for a short period of time [43]. Similarly, the larvae of *A. galli* have a tissue-associated phase [44]. Thus, at wpi 2, the hosts’ small intestine and caeca walls are mainly colonized by larvae, and almost no adult worms are present at this time, but by wpi 4, antibody response—as quantified by ELISA—decreases, coinciding with the presence of maturing worms in the lumen. This supports the idea that the larvae, which penetrate the intestinal wall of infected chickens, elicit a stronger humoral response than the adult worms that have migrated to the lumen [41]. Therefore, the migration of larvae from the intestinal walls to the lumen may have resulted in lower antibody production in subsequent weeks. However, at wpi 14, indicating the presence of the next generation of larvae due to re-infection, antibody response was again significantly higher in infected hens. The study by Marcos-Atxutegi et al. [41] established that soluble antigen from embryonated eggs stimulates a higher antibody concentration measured by ELISA than the adult worm antigen. Similarly, our results from the correlation between plasma IgY and worm stages further demonstrated that plasma IgY has a stronger positive relationship with the number of larvae than with adult worms. This result is also consistent with a previous report shown by Daş et al. [22]. This relationship was much stronger with *H. gallinarum* larvae than with *A. galli*, likely because of the higher re-infection with *H. gallinarum* than with *A. galli* [24].

As the chicken host develops cross-reactive antibodies against the two closely related nematode species [13], higher re-infection with *H. gallinarum* than with *A. galli* might explain higher correlations between ascarid-specific IgY and *H. gallinarum* larvae counts. Despite studying multiple time points, plasma IgY had no significant relationship with total worm burden, making it less suitable for quantitative diagnosis of nematode infection in chickens. However, in terms of qualitative diagnostic assessment, measuring ascarid-specific plasma IgY can be valuable considering its relatively high diagnostic accuracy, sensitivity and specificity obtained in this study. The sensitivity values ranged from 66 to 88% across all wpi. This value is lower when compared with previous studies. Sharma et al. [20] reported diagnostic sensitivity of 96% for plasma IgY in detecting *A. galli* infection in chickens, which was similar to the result reported by Daş et al. [13], who also reported sensitivity of 94%. As indicated in this study, the time of sample collection influences the diagnostic performance of the method used. Here, the diagnostic performance of the antibody ELISA was assessed continuously at different wpi up to wpi 18, unlike in the aforementioned studies where evaluation was made only at 25 wpi and 28 wpi, respectively, and may be responsible for the differences in results. Nevertheless, plasma IgY measurements can be considered a reliable tool for early detection of nematode infection in chickens.

By contrast, FEC, although not quantifiable until wpi 4 due to the pre-patent period [45, 46], shows a consistent and considerably stronger relationship with total worm burden. It is no surprise that FEC could not be evaluated until wpi 4, because egg counting in faeces relies on the presence of female worms with reproductive maturity in the host intestine, and it takes up to 5–8 weeks for larvae to reach maturity and begin shedding eggs, depending on several factors [45]. It is well known that FEC is a good reflection of infection intensity, but as shown in this study, the relationship differs based on the time of measurement. At wpi 4, FEC showed a low correlation with adult worms, whereas from wpi 6, FEC demonstrated a moderately high correlation with adult and total worm burden (Fig. 7b). Feyera et al. [47] found a similar range of correlation at wpi 8 and 10. As the eggs of the two nematode species cannot be reliably differentiated [29], the present correlations between EPG and worm burden of each species might also have been underestimated.

The ROC analysis showed that at wpi 2, coproantigen ELISA could not accurately differentiate between infected and non-infected hens at this early phase. Similarly, there was no significant difference in the faecal antigen concentration between the two groups at this time point. This is likely because the coproantigen ELISA is not sensitive enough to detect the possibly low concentration of antigens released by small larvae. However, from wpi 4 until the end of the experiment, coproantigen exhibited diagnostic sensitivity and specificity in the range of 61–100% and 90–100%, respectively. Further studies are needed to confirm whether worm antigen excreted in faeces is dependent on or associated with egg shedding. Such a study could evaluate changes in faecal antigen concentration in the presence or absence of worm eggs spiked in faeces. In general, the ROC analysis demonstrated higher qualitative performance for coproantigen ELISA than for IgY ELISA and FEC except in wpi 2, when plasma-IgY ELISA was more sensitive. The correlation between APG and total burden of *H. gallinarum* and *A. galli* was highest at wpi 4 and wpi 6, respectively. APG correlated best with *A. galli* weight rather than with the number of *A. galli* worms, implying the excretion of more antigens from the larger worms; however, data for *H. gallinarum* weight is not available for comparison. Based on the available data, it may be a reasonable assumption that faecal antigen concentration is more reflective of worm size than the number of the worms.

## Conclusion

We conclude that soluble worm antigens are consistently excreted through the faeces of an infected host and can be repeatedly measured on the same hen over time. In comparison with other methods, coproantigen ELISA provides the best qualitative diagnostic method. Plasma IgY assay is shown to be the most reliable tool for early diagnosis of nematode infection. Finally, in terms of infection intensity, FEC is superior, and remains a better indicator of total adult worm burden but only post-patency. We suggest that the combination of different tools rather than just one tool would give a better reflection of infections as a result of changes in developmental stage, worm size and fecundity over time. This suggests the necessity of complementary use of different tools for a more accurate diagnosis and quantification of infections.

## Supplementary Information

**Additional file 1: Figure S1.** Weight–length relationship of *A* *galli* male (a) and female (b) worms. https://doi.org/10.5281/zenodo.7974367.

**Additional file 2: Table S1.** Qualitative test performance parameters of different diagnostic tests derived from the receiver operator characteristics (ROC) analysis.

## Data Availability

Data used in this study have been deposited in an open-source data repository (DOI: 10.5281/zenodo.7974367).

## Abbreviations

APG: Antigen per gram of faeces
AUC: Area under the curve
ELISA: Enzyme-linked immunosorbent assay
EPG: Eggs per gram of faeces
FEC: Faecal egg count
ROC: Receiver operating characteristics
