# Supplementary figures and images for: Pattern and repeatability of ascarid-specific antigen excretion through chicken faeces, and the diagnostic accuracy of coproantigen measurements as compared with McMaster egg counts and plasma and egg yolk antibody measurements in laying hens
Source: Parasit Vectors. 2023 Jun 1;16:175. doi: 10.1186/s13071-023-05782-5 (PMC10234079; doi:10.1186/s13071-023-05782-5)

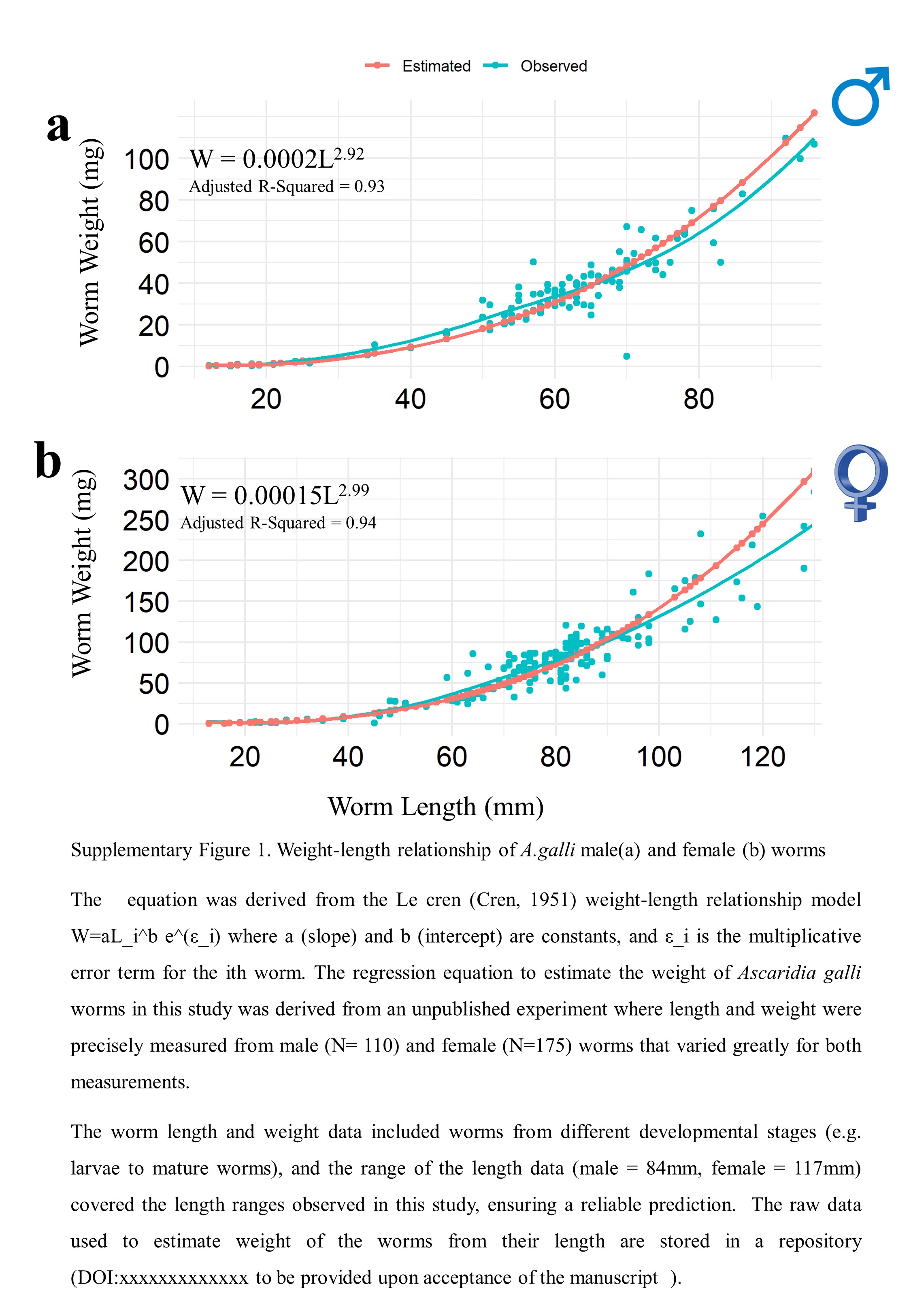

Supplement: Supplementary file 1 — Additional file 1: Figure S1. Weight–length relationship of A galli male (a) and female (b) worms. https://doi.org/10.5281/zenodo.7974367. [file 13071_2023_5782_MOESM1_ESM.jpg]
