# Supplementary material for: Pattern and repeatability of ascarid-specific antigen excretion through chicken faeces, and the diagnostic accuracy of coproantigen measurements as compared with McMaster egg counts and plasma and egg yolk antibody measurements in laying hens
Source: Parasit Vectors. 2023 Jun 1;16:175. doi: 10.1186/s13071-023-05782-5 (PMC10234079; doi:10.1186/s13071-023-05782-5)
Supplement: Supplementary file 2 — Additional file 2: Table S1. Qualitative test performance parameters of different diagnostic tests derived from the receiver operator characteristics (ROC) analysis. [file 13071_2023_5782_MOESM2_ESM.docx]

**Table S1.** Qualitative test performance parameters of different diagnostics tests derived from the receiver operator characteristics (ROC) analysis.

|  |  | **Diagnostic methods** | | | |
| --- | --- | --- | --- | --- | --- |
| **WPI** | Test performance parameters | Copro-antigen ELISA | FEC | Plasma IgY ELISA | Egg-Yolk IgY ELISA |
|  | N | 29 |  | 29 | 29 |
| **2** | AUC | 0.47 |  | 0.95 | 0.73 |
|  | Cut-off | 0.28 | NA | 46.85 | 11.4 |
|  | Sensitivity (%) | 88.9 |  | 88.90 | 50.0 |
|  | Specificity (%) | 0.0 |  | 100.0 | 80.0 |
|  |  |  |  |  |  |
|  | N | 29 | 29 | 29 | 29 |
| **4** | AUC | 0.76 | 0.69 | 0.83 | 0.98 |
|  | Cut-off | 0.28 | 12.50 | 46.85 | 11.4 |
|  | Sensitivity (%) | 39.0 | 38.9 | 66.7 | 100.0 |
|  | Specificity (%) | 100.0 | 100.0 | 90.9 | 82.0 |
|  |  |  |  |  |  |
|  | N | 29 | 29 | 29 | 29 |
| **6** | AUC | 0.96 | 0.94 | 0.65 | 0.76 |
|  | Cut-off | 0.28 | 12.5 | 46.85 | 11.4 |
|  | Sensitivity (%) | 72.0 | 88.9 | 16.7 | 78.0 |
|  | Specificity (%) | 100.0 | 100.0 | 90.9 | 73.0 |
|  |  |  |  |  |  |
|  | N | 29 | 29 | 29 | 29 |
| **10** | AUC | 0.97 | 0.97 | 0.88 | 0.90 |
|  | Cut-off | 0.28 | 12.5 | 46.85 | 11.4 |
|  | Sensitivity (%) | 88.9 | 94.4 | 44.4 | 71.0 |
|  | Specificity (%) | 100.0 | 100.0 | 90.9 | 89.0 |
|  |  |  |  |  |  |
|  | N | 28 | 28 | 28 | 28 |
| **14** | AUC | 0.95 | 0.97 | 0.92 | 0.84 |
|  | Cut-off | 0.28 | 12.5 | 46.85 | 11.4 |
|  | Sensitivity (%) | 83.3 | 94.4 | 44.4 | 76.0 |
|  | Specificity (%) | 100.0 | 100.0 | 100 | 80.0 |
|  |  |  |  |  |  |
|  | N | 34 | 34 | 34 | 34 |
| **18** | AUC | 1.00 | 0.97 | 0.88 | 0.92 |
|  | Cut-off | 0.28 | 12.5 | 46.85 | 11.4 |
|  | Sensitivity (%) | 100.0 | 94.4 | 61.1 | 81.0 |
|  | Specificity (%) | 100.0 | 100.0 | 100.0 | 79.0 |
|  |  |  |  |  |  |
| **All** | N* | 149 | 149 | 149 | 149 |
|  | AUC | 0.93^a^ | 0.91^a^ | 0.83^b^ | 0.88^a^ |
|  | Cut-off | 0.28 | 12.5 | 27.95 | 11.4 |
|  | Sensitivity (%) | 76.7 | 82.2 | 76.7 | 80.0 |
|  | Specificity (%) | 100.0 | 100.0 | 72.9 | 80.0 |

Birds were orally inoculated with either 1000 embryonated eggs of *Ascaridia galli* and *Heterakis gallinarum* eggs or with NaCl (control). All measurements including APG (using copro-antigen ELISA), EPG (using McMaster technique) and IgY in plasma and egg-yolks (using ascarid-specific IgY ELISA) were performed at different weeks post-infection (wpi; 2,4,6,10,14,18).

**Abbreviations**: N: number of observations; AUC: area under curve; APG: antigen concentration per gram faeces (µg/g); FEC: faecal egg counts; WPI: weeks post infection; NA: not applicable.

*Since EPG could not be estimated in wpi 2, only the data available from wpi 4 was used for the overall ROC analysis.
